# Clinicomolecular Profile and Efficacy of Human Epidermal Growth Factor Receptor 2 (HER2)-Targeted Therapy for *HER2*-Amplified Advanced Biliary Tract Cancer

**DOI:** 10.1200/PO-24-00718

**Published:** 2025-04-10

**Authors:** Kanae Inoue, Yoshiaki Nakamura, Bennett Caughey, Binbin Zheng-Lin, Makoto Ueno, Masayuki Furukawa, Yasuyuki Kawamoto, Shinji Itoh, Kumiko Umemoto, Kentaro Sudo, Taroh Satoh, Nobumasa Mizuno, Takeshi Kajiwara, Takao Fujisawa, Hideaki Bando, Takayuki Yoshino, John H. Strickler, Chigusa Morizane, Tanios Bekaii-Saab, Masafumi Ikeda

**Affiliations:** ^1^ Department of Hepatobiliary and Pancreatic Oncology, National Cancer Center Hospital East, Kashiwa, Japan; ^2^ Translational Research Support Office, Division of Drug and Diagnostic Development Promotion, Department for the Promotion of Drug and Diagnostic Development, National Cancer Center Hospital East, Kashiwa, Japan; ^3^ Department of Gastroenterology and Gastrointestinal Oncology, National Cancer Center Hospital East, Kashiwa, Japan; ^4^ Division of Hematology/Oncology, Massachusetts General Hospital, Boston, MA; ^5^ Division of Hematology and Medical Oncology, Mayo Clinic, Phoenix, AZ; ^6^ Department of Gastroenterology, Kanagawa Cancer Center, Yokohama, Japan; ^7^ Department of Hepato-Biliary-Pancreatology, National Hospital Organization Kyushu Cancer Center, Fukuoka, Japan; ^8^ Division of Cancer Center, Hokkaido University Hospital, Sapporo, Japan; ^9^ Department of Surgery and Science, Graduate School of Medical Sciences, Kyushu University, Fukuoka, Japan; ^10^ Department of Clinical Oncology, St Marianna University School of Medicine, Kawasaki, Japan; ^11^ Department of Gastroenterology, Chiba Cancer Center, Chiba, Japan; ^12^ Center for Cancer Genomics and Precision Medicine, Osaka University Hospital, Osaka, Japan; ^13^ Department of Gastroenterology, Aichi Cancer Center Hospital, Nagoya, Japan; ^14^ Department of Gastrointestinal Medical Oncology, National Hospital Organization Shikoku Cancer Center, Matsuyama, Japan; ^15^ Department of Head and Neck Medical Oncology, National Cancer Center Hospital East, Kashiwa, Japan; ^16^ Duke Cancer Institute, Duke University, Durham, NC; ^17^ Department of Hepatobiliary and Pancreatic Oncology, National Cancer Center Hospital, Tokyo, Japan

## Abstract

**PURPOSE:**

This study aimed to investigate the clinicomolecular profiles and the efficacy of human epidermal growth factor receptor 2 (HER2)-targeted therapy in *HER2*-amplified biliary tract cancer (BTC).

**METHODS:**

This study was an international collaboration that used combined data from the prospective SCRUM-Japan GOZILA and MONSTAR-SCREEN in Japan and retrospective reviews in the United States; patients with advanced BTC who had received systemic therapy were included. The clinicomolecular profiles were evaluated in an exploratory cohort, whereas the efficacy of HER2-targeted therapy was assessed in a biomarker-selected cohort.

**RESULTS:**

Of the 439 patients in the exploratory cohort, 43 (10%) had *HER2* amplification. The frequencies of coalterations were higher in patients with *HER2* amplification versus patients without *HER2* amplification including *HER2* mutations (26% *v* 5%, *P* < .001), *TP53* mutations (84% *v* 61%, *P* = .003), and *BRAF* amplification (9% *v* 2%, *P* = .030). There were no *KRAS* mutations identified in patients with *HER2-*amplified BTC. No significant difference in overall survival (OS) was observed between patients with and without *HER2* amplification (median, 17.7 *v* 16.9 months; hazard ratio [HR], 0.95 [95% CI, 0.65 to 1.40]). Of the 60 patients with *HER2*-amplified BTC in the biomarker-selected cohort (43 from Japan and 17 from the United States), the OS was significantly longer in 29 patients who received HER2-targeted therapy than in those who did not receive HER2-targeted therapy (median, 24.3 *v* 12.1 months; HR, 0.39 [95% CI, 0.23 to 0.82]). Multivariate analysis identified HER2-targeted therapy as an independent prognostic factor for OS (HR, 0.29 [95% CI, 0.14 to 0.58]; *P* < .001).

**CONCLUSION:**

*HER2* amplification was found in 10% of advanced BTC and was not identified as an independent prognostic factor for OS. Patients with *HER2*-amplified BTC derive significant benefit from HER2-targeted therapy.

## BACKGROUND

Biliary tract cancer (BTC) includes intrahepatic and extrahepatic cholangiocarcinoma, gallbladder cancer, and cancer of the ampulla of Vater. BTC is typically diagnosed at an advanced stage and carries a poor prognosis.^1^ First-line systemic therapy for advanced BTC includes gemcitabine plus cisplatin (GC) with or without durvalumab or pembrolizumab.^2-4^ GC plus S-1 (an oral fluoropyrimidine) also improved overall survival (OS) over GC alone and is one of the standard first-line treatments in Japan.^5^ However, the median OS of patients receiving these treatments is approximately 12 months, and second-line treatment options such as fluorouracil, leucovorin, and oxaliplatin, and nanoliposomal irinotecan in combination with fluorouracil and leucovorin show limited efficacy and provide modest clinical benefit.^6,7^ S-1 monotherapy is often used in Japan on the basis of an objective response rate (ORR) of 7.5%-22.7%; however, it has not been shown to improve OS.^8,9^ Therefore, additional effective treatments are needed for patients with advanced BTC after first-line treatment.

**CONTEXT**

**Key Objective**
What is the comprehensive clinicomolecular profile of human epidermal growth factor receptor 2 (*HER2*)–amplified biliary tract cancer (BTC) and does HER2-targeted therapy improve the treatment outcome in this population?
**Knowledge Generated**
Our international collaborative study revealed that *HER2* amplifications were found in 10% of patients with advanced BTC, and that *HER2* amplification was not an independent prognostic factor of overall survival (OS). HER2-targeted therapy demonstrated significant improvement of OS in this population.
**Relevance**
This study showed that treatment targeting HER2 provided survival benefit in patients with advanced BTC, which strongly supports the need for continued efforts to develop HER2-targeted therapies for patients with *HER2*-amplified advanced BTC.

Patients harboring specific molecular aberrations could derive benefit from targeted therapies in the second- or later-line setting.^10-15^ Human epidermal growth factor receptor 2 (HER2) is a tyrosine kinase transmembrane receptor, and its overexpression/amplification, which has been identified as an oncogenic driver in multiple malignancies, is associated with a poor prognosis in some cancers. HER2-targeted therapy has been demonstrated to be effective for advanced lung, breast, gastric, and colorectal cancers, and is a standard treatment for these cancers.^16-26^ The efficacy of trastuzumab deruxtecan has also been demonstrated to be effective across HER2-expressing solid tumors.^27^ The reported frequency of *HER2* amplification in patients with advanced BTC is 5%^28-30^; however, BTC is anatomically heterogeneous and the reported frequency of *HER2* amplification/overexpression varies by primary tumor site: 11%-18.5% in extrahepatic cholangiocarcinoma, 3%-4.8% in intrahepatic cholangiocarcinoma, 16%-31.3% in gallbladder cancer, and 13%-27.9% in cancer of the ampulla of Vater.^31-36^ Development of HER2-targeted therapy for BTC is ongoing, and several phase II trials have been conducted, with a reported ORR of 23.0%-46.7%, progression-free survival (PFS) of 4.0-5.5 months, and OS of 7.1-15.5 months.^37-41^ However, the survival benefit of HER2-targeted therapy in patients with advanced BTC remains unclear. Furthermore, there are only a few reports on the clinicomolecular profile of *HER2-*amplified or overexpressing BTC despite the necessity to understand it for further therapeutic development.^28,42^

In an international collaboration between multiple centers in Japan and four centers in the United States (Mayo Clinic in Arizona, Florida, and Minnesota, and Duke University in North Carolina), we investigated the comprehensive clinicomolecular profiles of *HER2*-amplified BTC and the efficacy of HER2-targeted therapy in *HER2*-amplified BTC.

## METHODS

### Patients and Study Design

Patients with advanced BTC from the SCRUM-Japan GOZILA and MONSTAR-SCREEN studies in Japan and from clinically annotated retrospective chart reviews in the United States who had received systemic therapy were included in this study. The inclusion criteria were as follows: (1) advanced BTC diagnosed by histopathology or cytology; (2) age ≥18 years; (3) history of palliative systemic therapies; and (4) comprehensive genomic profiling performed, and maximum variant allele frequency (VAF) of ≥1 if circulating tumor DNA (ctDNA) analysis was performed. Genomic alterations were detected by tissue next-generation sequencing (NGS) using FoundationOne CDx (Foundation Medicine) or Tempus xT (Tempus, Chicago, IL), and plasma ctDNA NGS using Guardant360 CDx (Guardant Health, Palo Alto, CA) or FoundationOne Liquid CDx (Foundation Medicine, Boston, MA), and reported using their clinical thresholds. The adjusted plasma copy number (pCN) of *HER2* was calculated as follows: adjusted pCN = (observed pCN – 2 × [1 – T%])/T%, where T% = 2 × maximum VAF/100.^43^ pCN-high and adjusted pCN-high were defined as values higher than the median, and pCN-low and adjusted pCN-low were defined as values lower than the median.

The GOZILA study enrolled patients with advanced solid tumors, including BTC, who showed disease progression during/after systemic therapy, and plasma NGS analysis was performed using Guardant360 at Guardant Health. The MONSTAR-SCREEN study enrolled patients with advanced solid tumors, including BTC, and plasma NGS analysis was performed using FoundationOne Liquid CDx and tissue NGS analysis was performed using FoundationOne CDx at Foundation Medicine. These study protocols were approved by the institutional review board at each institution. The studies were conducted in accordance with the protocols and in conformity with the principles of the Declaration of Helsinki. All patients provided written informed consent before being enrolled in the studies.

This study consisted of two cohorts: the exploratory cohort and the biomarker-selected cohort. In the exploratory cohort, composed of patients from Japan, we conducted a comparative evaluation of the clinicomolecular profiles of *HER2*-amplified BTC and non–*HER2*-amplified BTC. In the biomarker-selected cohort, composed of patients with *HER2-*amplified BTC from both Japan and the United States, we evaluated the efficacy of HER2-targeted therapy for *HER2*-amplified BTC.

### Data Collection and Definitions

In the exploratory cohort, the following individual patient data were collected: age, sex, primary tumor site, timing of NGS, type of NGS assay, site of metastasis, first-line treatment, genomic alterations, and OS. In the biomarker-selected cohort, the following additional data were collected: treatment line, regimen, PFS, and best overall response of HER2-targeted therapy. OS was defined as the time from the initiation of first-line treatment to death from any cause. PFS of HER2-targeted therapy was defined as the time from initiation of the HER2-targeted therapy to disease progression or death from any cause. ORR was defined as the proportion of patients who showed complete response (CR) or partial response (PR). The disease control rate (DCR) was defined as the proportion of patients who showed CR, PR, or stable disease (SD) as the best treatment response.

### Statistical Analysis

Categorical variables were compared by using Fisher's exact test, while continuous variables were compared by using the Mann-Whitney *U* test. The OS and PFS were analyzed by using the Kaplan-Meier method and compared by using the log-rank test. Multivariate analysis was conducted using the Cox proportional hazards model. *P* values <.05 were considered statistically significant. All reported *P* values are two-sided. All the statistical analyses were conducted using R version 3.4.1 (R Foundation for Statistical Computing, Vienna, Austria).

## RESULTS

### Characteristics of the Patients

**FIG 1.**
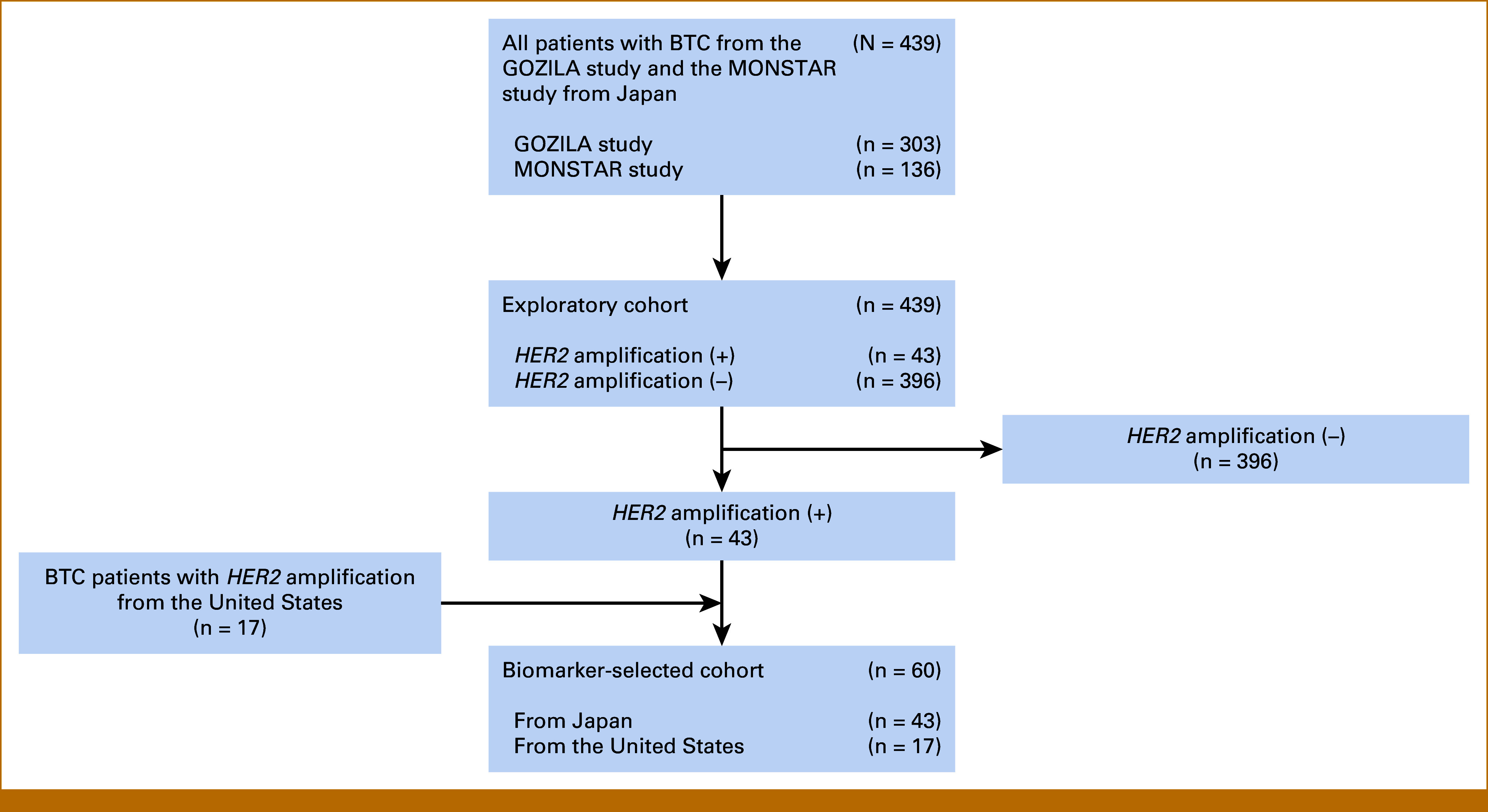
Study design. The exploratory cohort comprised 439 patients, including 303 patients from the GOZILA study and 136 patients from the MONSTAR-SCREEN study in Japan. The biomarker-selected cohort comprised 60 patients with *HER2* amplification, including 43 patients from Japan and 17 patients from the United States. BTC, biliary tract cancer; HER2, human epidermal growth factor receptor 2.

The exploratory cohort consisted of 439 patients, including 303 patients from 31 institutions participating in the GOZILA study (registered from September 2018 to December 2022) and 136 patients from 31 institutions participating in the MONSTAR-SCREEN study (registered from April 2020 to March 2022) in Japan. The biomarker-selected cohort consisted of 43 patients from Japan and 17 patients extracted from a retrospective chart review at four centers in the United States, all of who had *HER2* amplification (Fig 1). The characteristics of the patients in the exploratory cohort are summarized in Table 1. Of the 439 patients in the exploratory cohort, 43 (10%) had *HER2* amplification. A higher percentage of patients with *HER2* amplification, compared with those without *HER2* amplification, were female (67% *v* 38%, *P* < .001) and had gallbladder cancer (58% *v* 29%, *P* < .001), with no significant differences in other characteristics between the two groups. The cohort included 36 patients who had received HER2-targeted therapy, including 17 patients with *HER2* amplification and 19 patients without *HER2* amplification.

**TABLE 1.** Patient Characteristics of the Patients in the Exploratory Cohort

Characteristic	*HER2* Amplification (+) (n = 43)	*HER2* Amplification (–) (n = 396)	*P*
Age, years, median (range)	67.0 (39-79)	69.0 (28-84)	.232
Sex, No. (%)			<.001
Male	14 (33)	244 (62)	
Female	29 (67)	152 (38)	
Primary site, No. (%)			<.001
Intrahepatic	5 (12)	144 (36)	
Extrahepatic	5 (12)	92 (23)	
Gallbladder	25 (58)	122 (29)	
Ampulla	8 (18)	48 (12)	
NGS assay, No. (%)			.565
Plasma	41 (95)	360 (91)	
Tissue	2 (5)	36 (9)	
Test timing of NGS before second-line treatment, No. (%)	13 (30)	98 (25)	.719
No. of metastatic organs ≥2	21 (49)	158 (40)	.421
Site of metastasis, No. (%)			
Liver	29 (67)	211 (53)	.106
Lung	15 (35)	85 (21)	.056
Peritoneum	5 (12)	63 (16)	.657
Lymph node	24 (56)	173 (44)	.147
Other	1 (2)	41 (10)	.104
First-line treatment, No. (%)			.567
GC	30 (70)	230 (58)	
GCS	6 (14)	86 (22)	
GS	1 (2)	19 (5)	
Other	6 (14)	61 (15)	
HER2-targeted therapy	17 (40)	19 (5)	<.001

Abbreviations: GC, gemcitabine + cisplatin; GCS, gemcitabine + cisplatin + S-1; GS, gemcitabine + S-1; HER2, human epidermal growth factor receptor 2; NGS, next-generation sequencing.

### Genomic Alterations

**FIG 2.**
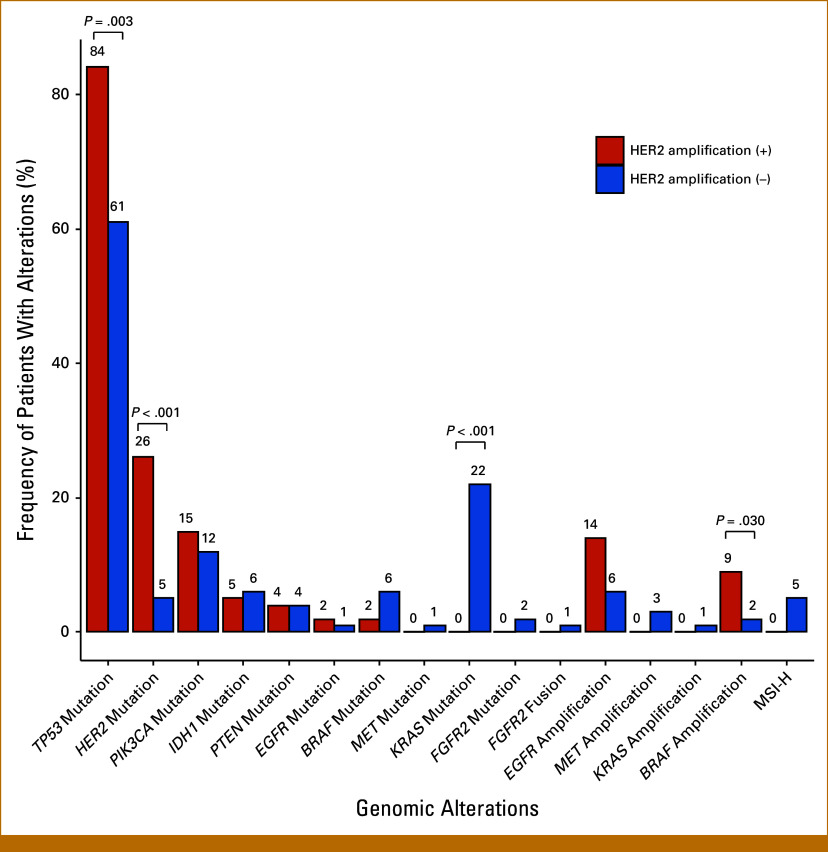
Genomic alterations in patients with and without *HER2* amplification. The frequencies of coalterations were higher in patients with *HER2* amplification versus patients without *HER2* amplification, including *HER2* mutations (26% *v* 5%, *P* < .001), *TP53* mutations (84% *v* 61%, *P* = .003), and *BRAF* amplification (9% *v* 2%, *P* = .030). *KRAS* mutations were found in 22% of patients without *HER2* amplification, but no *KRAS* mutations were found in patients with *HER2* amplification. HER2, human epidermal growth factor receptor 2; HR, hazard ratio; OS, overall survival.

The genomic alterations in the exploratory cohort are shown in Figure 2. Coalterations were more frequent in patients with *HER2* amplification than without, including *HER2* mutations (26% *v* 5%, *P* < .001), *TP53* mutations (84% *v* 61%, *P* = .003), and *BRAF* amplification (9% *v* 2%, *P* = .030). *KRAS* mutations were found in 22% of patients without HER2 amplification but were absent in those with *HER2* amplification (*P* < .001).

### Survival Outcomes

**FIG 3.**
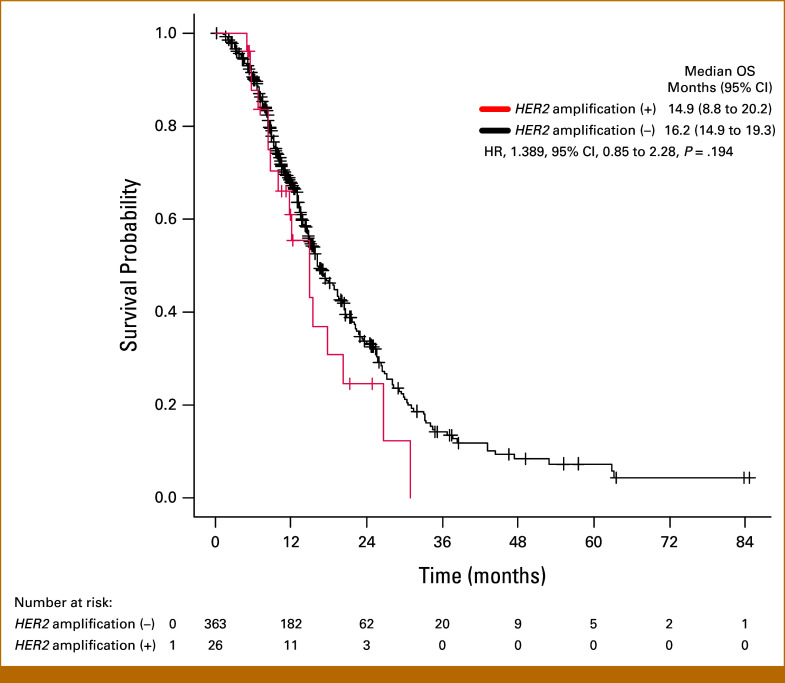
Kaplan-Meier curves of OS in patients with and without *HER2* amplification, excluding patients who received HER2-targeted therapy. After excluding 36 patients who received HER2-targeted therapy, among the 26 patients with *HER2* amplification, events occurred in 17 patients; among the 363 patients without *HER2* amplification, events occurred in 223 patients. The median OS was 14.9 months (95% CI, 8.8 to 20.2) in the patients with *HER2* amplification and 16.2 months (95% CI, 14.9 to 19.3) in those without *HER2* amplification. HER2, human epidermal growth factor receptor 2; OS, overall survival.

Of the 439 patients in the exploratory cohort, OS data were available for 425 patients and the survival analysis was performed on the basis of 268 events (63%). The median OS was 17.7 months (95% CI, 11.8 to 24.6) in patients with *HER2* amplification and 16.9 months (95% CI, 15.6 to 19.3) in patients without *HER2* amplification, with no significant difference between the two groups (hazard ratio [HR], 0.95 [95% CI, 0.65 to 1.40]; *P* = .799). Among the four groups stratified by *HER2* amplification status and HER2-targeted therapy, the HER2-amplified group with HER2-targeted therapy had the longest OS, with a median of 21.5 months (Data Supplement, Fig S1). Among 425 patients, an analysis focusing on the 389 patients who had not received HER2-targeted therapy was conducted (excluding the 36 patients who had received HER2-targeted therapy). The median OS was 14.9 months in patients with *HER2* amplification and 16.2 months in patients without *HER2* amplification, with no significant difference between the two groups (HR, 1.39 [95% CI, 0.85 to 2.28]; *P* = .194; Fig 3).

The prognostic factors for OS of patients in the exploratory cohort are shown in the Data Supplement (Fig S2). Multivariate analysis identified the test timing of NGS before second-line treatment (HR, 1.82 [95% CI, 1.38 to 2.40]; *P* < .001), liver metastasis (HR, 1.35 [95% CI, 1.03 to 1.70]; *P* = .027), and peritoneal dissemination (HR, 1.46 [95% CI, 1.04 to 2.10]; *P* = .028) as independent poor prognostic factors for OS. However, *HER2* amplification was not identified as an independent prognostic factor for OS (HR, 1.01 [95% CI, 0.66 to 1.50]; *P* = .958).

### *HER*2-Amplified BTC

The characteristics of the patients in the biomarker-selected cohort are summarized in the Data Supplement (Table S1). Of the 60 patients with *HER2*-amplified BTC in this cohort, 43 (72%) patients were determined by plasma NGS as harboring *HER2* amplification. Tissue NGS was performed by FoundationOne CDx in 12 patients and Tempus xT in five patients, and plasma NGS was performed by Guardant360 CDx in 34 patients and FoundationOne Liquid CDx in nine patients. The median tissue CN was 12.5 (range, 4-109), the median pCN was 8.9 (range, 2.3-154.0), and the adjusted pCN was 25.7 (range, 2.4-2,063.4; Data Supplement, Fig S3). Of the *HER2*-amplified BTC patients, 29 (48%) received HER2-targeted therapy, including 18 from Japan and 11 from the United States. NGS was performed before the initiation of HER2-targeted therapy in 25 patients (86%). The characteristics of the patients with *HER2*-amplified BTC by country are summarized in the Data Supplement (Table S2). Tissue NGS was performed more commonly in the United States, while plasma NGS was performed more commonly in Japan.

### Efficacy of HER2-Targeted Therapy

**FIG 4.**
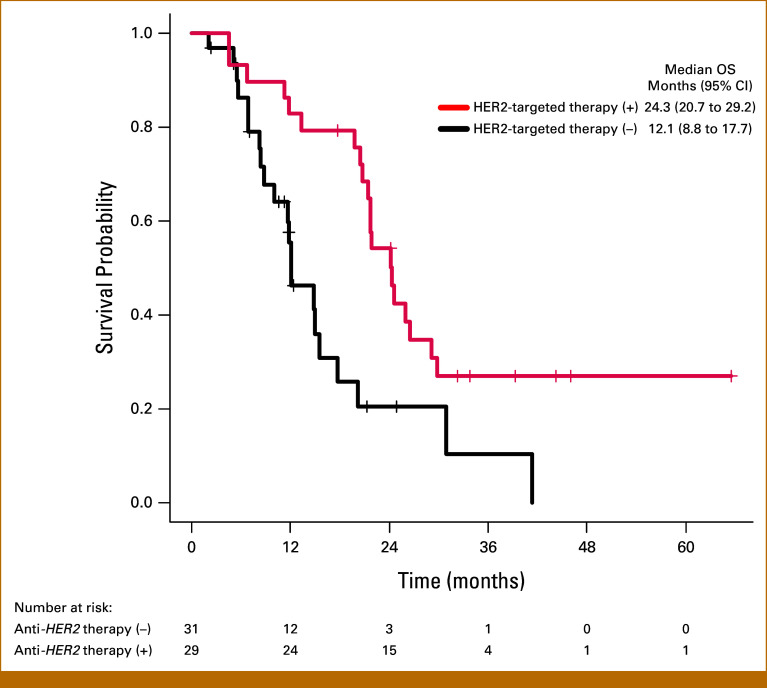
Kaplan-Meier curves of OS in patients with *HER2* amplification who received/did not receive HER2-targeted therapy. The median OS was 24.3 months (95% CI, 20.7 to 29.3) in the patients who received HER2-targeted therapy and 12.1 months (95% CI, 8.8 to 17.7) in those who did not receive HER2-targeted therapy. HER2, human epidermal growth factor receptor 2; OS, overall survival.

The details of the HER2-targeted therapy received by 29 patients in the biomarker-selected cohort are summarized in the Data Supplement (Table S3). The median OS of the patients who received HER2-targeted therapy was 24.3 months, which was significantly longer than that in the patients who did not receive HER2-targeted therapy (12.1 months; HR, 0.39 [95% CI, 0.23 to 0.82]; *P* = .011; Fig 4). PFS data were available for 24 patients. The median PFS of the patients who received HER2-targeted therapy was 6.6 months (95% CI, 4.2 to 7.1 months). The best tumor responses were as follows: CR, 0 patient; PR, 10 patients (35%); SD, 12 patients (41%); PD, four patients (14%); and not evaluated, three patients (10%). The ORR and DCR were 34% and 76%, respectively. Of the 19 patients receiving HER2-targeted therapy who underwent plasma NGS, 17 patients, excluding two with missing CN, were analyzed for PFS on the basis of pCN and adjusted pCN status. There was no difference in PFS between patients classified as *HER2* (adjusted) pCN-high and *HER2* (adjusted) pCN-low (Data Supplement, Fig S4). Among 10 patients who underwent tissue NGS, CN data were available for only five, so no analysis was performed. Regarding coalterations and PFS, patients with *BRAF* amplification (n = 2) had shorter PFS than those without (n = 22), although not statistically significant (3.5 *v* 6.8 months; HR, 5.15 [95% CI, 0.93 to 28.36]; *P* = .060). OS data were available for 60 patients with *HER2* amplification in the biomarker-selected cohort and the survival analysis was performed on the basis of 41 events (68%). The prognostic factors for OS in patients with *HER2* amplification are shown in the Data Supplement (Fig S5). Multivariate analysis identified the test timing of NGS before second-line treatment (HR, 3.52 [95% CI, 1.68 to 7.39]; *P* < .001) and liver metastasis (HR, 2.56 [95% CI, 1.06 to 6.18]; *P* = .037) as poor prognostic factors, and HER2-targeted therapy (HR, 0.29 [95% CI, 0.14 to 0.58]; *P* < .001) as an independent favorable prognostic factor for OS.

## DISCUSSION

Several small clinical trials have shown promising efficacy of HER2-targeted therapy in patients with *HER2*-amplified BTC; however, its impact on OS remains unclear. Our international collaborative study revealed the clinicomolecular profiles of patients with *HER2*-amplified BTC, and also demonstrated that HER2-targeted therapy significantly improved OS of this population. This benefit of HER2-targeted therapy was maintained even after multivariate analysis with adjustments for confounding factors. This finding strongly suggests the need for continued efforts to develop HER2-targeted therapies for patients with *HER2*-amplified advanced BTC.

The frequency of *HER2* amplification in BTC varies according to the primary tumor site, and is reported to be highest in gallbladder cancer and lowest in intrahepatic cholangiocarcinoma,^28,31,36^ which was confirmed in our study (3% in intrahepatic cholangiocarcinoma, 5% in extrahepatic cholangiocarcinoma, 27% in gallbladder cancer, and 14% in cancer of the ampulla of Vater). A high concordance rate between the results of plasma NGS and tissue NGS to detect *HER2* amplification in advanced BTC has been reported, with a detection frequency of 5% by both assays.^30^ In the exploratory cohort in our study, plasma NGS was used in 401 patients (91%) and tissue NGS in 38 patients (9%); *HER2* amplification was detected in 41 of 401 patients (10.2%) by plasma NGS and 2 of 38 patients (5.2%) by tissue NGS.

It has been reported that *TP53*, *HER3*, and *PIK3CA* mutations are more common, and *KRAS* mutations are less common in *HER2*-amplified BTC.^28^ This study found significantly higher frequencies of *HER2* mutations, *TP53* mutations, and *BRAF* amplification in patients with *HER2* amplification, and no *KRAS* mutations were observed.

Results of previous studies of the prognostic role of HER2 overexpression and *HER2* alterations in patients with BTC have been inconclusive. A retrospective study found that HER2-positive patients had shorter disease-free survival and OS compared with HER2-negative patients, although these differences were not statistically significant.^44^ However, HER2-positive patients in that study also had worse performance status and received less adjuvant chemotherapy, potentially confounding the results. Another study found HER2 alterations in 14.9% of 121 patients with advanced BTC, with no significant difference in the response rate to GC therapy between patients with and without *HER2* alterations. In addition, *HER2* alterations were not identified as an independent prognostic factor for PFS or OS.^42^ Consistent with the finding of the latter study, in our study, *HER2* amplification was not identified as an independent prognostic factor for OS in patients with advanced BTC.

In the biomarker-selected cohort, which included only patients with *HER2-*amplified BTC, 29 patients received HER2-targeted therapy, including 18 patients from Japan and 11 patients from the United States. As for the efficacy of HER2-targeted therapy, the ORR and DCR were 34% and 76%, respectively, which were comparable with the results of each of the phase II trials (ORR, 23.0%-46.7%; DCR, 51.0%-81.8%).^37-41^ Although there was no significant difference in the patient characteristics between patients who did or did not receive HER2-targeted therapy, the OS was significantly longer in the patients who received HER2-targeted therapy. Furthermore, both univariate and multivariate analyses identified HER2-targeted therapy as an independent favorable prognostic factor for OS. Among the phase II trials of HER2-targeted therapy for advanced BTC, patient selection was based on different selection criteria, and the most appropriate modality of HER2 testing (eg, immunohistochemistry [IHC], in situ hybridization, or NGS) has not yet been determined. This study demonstrated a significant prognostic benefit of HER2-targeted therapy in patients with *HER2*-amplified BTC, and it is hoped that HER2-targeted therapy will be approved for *HER2*-amplified BTC in the future. Although higher *HER2* copy numbers have been reported to be associated with a better response to HER2-targeted therapy in other cancers,^43,45^ no difference in PFS was observed in our study between patients with and without *HER2* CN-high. Reactivation of the HER2 pathway or its downstream signaling, including coalterations in RTK/RAS/PI3K pathways, has been reported as a mechanism of resistance to HER2-targeted therapy.^45-47^ Two HER2-amplified BTC patients with BRAF amplification showed shorter PFS compared with those without, although the small sample size limits statistical reliability. BRAF amplification, reported to be associated with poor prognosis,^48,49^ may explain this finding. Therefore, we analyzed the OS of patients with and without *BRAF* amplification in the exploratory cohort, but there were only 13 patients with *BRAF* amplification, and no significant difference in OS was observed. Thus, the impact of *BRAF* amplification in patients with *HER2*-amplified BTC and HER2-targeted therapy in these patients need further investigation.

The test timing of NGS before second-line treatment was also identified as an independent poor prognostic factor for OS in both the study cohorts. This result is likely to be explained by the fact that only patients who underwent NGS were included in this study, and therefore, those who could be tested later in treatment line had a longer OS and better prognosis. This immortal time bias was demonstrated in the SCRUM-Japan GI-SCREEN, a large-scale genomic profiling program for patients with advanced gastrointestinal cancers using a validated genomic assay.^50^

Our study had several limitations. First, since most NGS assays in the exploratory cohort were plasma NGS assays and the concordance with tissue NGS assay was not confirmed, it is unclear if similar results would have been obtained if tissue NGS had been performed in most patients. Nevertheless, as discussed above, the *HER2* amplification status determined by plasma NGS has mostly been reported as being consistent with that determined by tissue NGS; therefore, we used tissue and plasma NGS data together. Second, HER2 expression was not evaluated by IHC in this study, although the IHC-HER2 status is mostly known to be concordant with the presence of *HER2* amplification, and NGS is widely used to identify *HER2*-amplified BTC for therapeutic decision making. Finally, because of lack of data, we could not evaluate the contribution of the performance status, a factor considered as being important in many studies evaluating the survival, in this study.

In conclusion, *HER2* amplifications were found in 10% of advanced BTC, and *HER2* amplification was not an independent prognostic factor for OS. Of clinical significance, patients with *HER2*-amplified BTC derive significant benefit from HER2-targeted therapy. To the best of our knowledge, this is the first study to show a survival benefit obtained with treatment targeting *HER2* in patients with advanced BTC.

## Data Availability

A data sharing statement provided by the authors is available with this article at DOI https://doi.org/10.1200/PO-24-00718. For GOZILA and MONSTAR-SCREEN, primary analyses are yet to be completed. Upon completion of these analyses, genomic data will be deposited in a genomic database. To protect the privacy and confidentiality of patients in this study, clinical data were not made publicly available in a repository or in the supplementary material of the article, but they will be available upon reasonable request to the corresponding author. The requests will be reviewed by a study steering committee to verify whether the request is subject to any intellectual property or confidentiality obligations. All data shared will be deidentified.
